# Analysis of Determinants and Development of a Predictive Model for Postoperative Cognitive Dysfunction in Patients Undergoing Hepatectomy

**DOI:** 10.3390/jcm15093508

**Published:** 2026-05-03

**Authors:** Yan Li, Jiawei Xu, Bing Xue, Jiahui Cao, Hanqi Yang, Xianwen Li

**Affiliations:** 1 School of Nursing, Nanjing Medical University, Nanjing 211103, China; manli131@163.com; 2 Department of Hepatobiliary Surgery, Nanjing DrumTower Hospital, Nanjing 210008, China; xjwnju0910@126.com (J.X.); 15651886320@163.com (B.X.); 18851863696@163.com (J.C.); 15501649032@163.com (H.Y.)

**Keywords:** postoperative cognitive dysfunction, nomogram, hepatectomy, risk prediction, perioperative factors

## Abstract

**Purpose:** This retrospective study aimed to identify factors associated with postoperative cognitive dysfunction (POCD) in patients undergoing hepatectomy, with particular attention to liver disease-related characteristics and perioperative variables. A secondary aim was to develop a clinically applicable nomogram for individualized risk estimation in this population. **Patients and Methods:** A retrospective cohort study was conducted in 314 consecutive patients who underwent hepatectomy at Nanjing Drum Tower Hospital Affiliated to Nanjing University Medical School between January 2023 and December 2024. Patients were included if they had complete clinical data and underwent preoperative and postoperative cognitive assessment. Exclusion criteria included preoperative cognitive impairment (Montreal Cognitive Assessment [MoCA] score < 26), preexisting neurological or psychiatric disorders, and in-hospital death within 72 h after surgery. POCD was defined as a decline of ≥3 points in the MoCA score from baseline to postoperative day 5. Clinical, surgical, nutritional, and perioperative variables were analyzed, and a nomogram was constructed based on the final multivariable logistic regression model. **Results:** The overall incidence of POCD was 27.4% (86/314). The final multivariable model included sarcopenia, preoperative hemoglobin < 120 g/L, Child–Pugh classification, alcohol consumption, operative duration, and pain score on postoperative day 1. The nomogram incorporating these variables showed good discriminative ability, with an area under the curve of 0.87 (95% CI: 0.83–0.92). **Conclusions:** In this retrospective cohort of patients undergoing hepatectomy, several perioperative clinical factors were associated with POCD. The proposed nomogram may serve as a practical tool for perioperative risk estimation and support more individualized management in higher-risk patients.

## 1. Introduction

Hepatic hemangioma, focal nodular hyperplasia, hepatocellular carcinoma, and intrahepatic cholangiocarcinoma are among the most common hepatic lesions in China. Surgical resection remains a primary therapeutic option for these conditions, but it is associated with postoperative complications such as hemorrhage, infection, biliary leakage, and postoperative cognitive dysfunction (POCD), all of which may substantially impair patients’ quality of life [1]. Reported within the first postoperative week after hepatectomy, POCD is characterized by impairments in memory, attention, and executive function, and may adversely affect postoperative recovery while also increasing the long-term risk of dementia [2,3]. Accordingly, POCD has become an important topic in perioperative research.

In recent years, postoperative neurocognitive complications have drawn increasing attention because they are not only common after major surgery but are also closely linked to subsequent recovery trajectories. Even when cognitive decline is transient, it may interfere with early mobilization, communication, treatment adherence, and rehabilitation planning. In the setting of hepatobiliary surgery, these consequences may be particularly relevant because recovery after hepatectomy often requires careful coordination of liver function monitoring, nutritional support, pain control, and prevention of postoperative complications. Therefore, early identification of patients with a higher likelihood of postoperative cognitive dysfunction may have practical value for perioperative management.

Beyond its direct effects on cognition, POCD is also associated with adverse postoperative outcomes. Patients who develop POCD may experience higher rates of postoperative complications, prolonged hospitalization, increased medical costs, and impaired long-term functional status and quality of life [4,5]. In patients with liver disease, the clinical implications of POCD may be particularly important. First, hyperammonemia resulting from hepatic insufficiency shares neurotoxic pathways with POCD and may overlap with or aggravate hepatic encephalopathy. Second, postoperative cognitive impairment may reduce treatment adherence and thereby increase the risk of severe complications such as post-hepatectomy liver failure and intra-abdominal infection [6,7]. Taken together, these considerations suggest that POCD after hepatectomy should not be viewed merely as an isolated neurological event, but rather as a clinically meaningful component of the overall postoperative recovery process.

Although factors associated with POCD have been widely investigated in elderly and cardiac surgery populations, they remain insufficiently characterized in patients undergoing hepatectomy. This population often presents with hepatic dysfunction, malnutrition, and chronic pain, all of which may contribute to postoperative cognitive vulnerability through inflammatory cascades, metabolic dysregulation, and neuroinflammation [8,9,10,11,12,13]. In addition, hepatectomy itself may impose substantial physiological stress through liver dysfunction, surgical trauma, fluid shifts, anesthetic exposure, and postoperative recovery burden. These features distinguish hepatectomy from many other major operations and suggest that findings derived from non-hepatic surgical cohorts may not be directly generalizable to this setting.

From a clinical standpoint, several perioperative domains may be relevant when evaluating cognitive outcomes after hepatectomy [14,15]. Baseline hepatic functional reserve may affect metabolic stability and susceptibility to postoperative neurotoxicity [16,17]. Nutritional impairment and sarcopenia may reflect reduced physiological reserve and impaired stress tolerance. Hematologic status, including preoperative anemia, may influence perioperative oxygen delivery to vulnerable tissues. Operative burden, reflected by resection extent or operative duration, may affect the magnitude of inflammatory and hemodynamic stress [18]. In addition, early postoperative pain and medication exposure may further shape short-term neurocognitive recovery. However, the relative contributions of these perioperative variables remain incompletely understood, and few studies have examined them within a unified analytical framework in patients undergoing hepatectomy.

In recent years, increasing attention has been directed toward identifying clinically relevant variables associated with POCD and developing practical tools for perioperative risk stratification. Predictive models that integrate multiple routinely available clinical factors may help support individualized perioperative management. However, the contributions of liver functional status, nutritional impairment, and operative burden to POCD after hepatectomy remain incompletely understood. Moreover, few studies have developed quantitative risk assessment tools specifically for patients undergoing hepatectomy, a population with distinct metabolic and physiological characteristics. A clinically accessible model based on routinely collected perioperative variables may therefore be useful for identifying patients who require closer surveillance and more individualized management during the perioperative period.

To address these knowledge gaps, we retrospectively analyzed data from 314 patients who underwent hepatectomy. The aims of this study were to evaluate the association of multidimensional perioperative variables, including liver function, nutritional status, and surgical stress, with POCD, and to develop an internally validated visual nomogram to support individualized perioperative risk estimation.

## 2. Methods

### 2.1. Participants

A retrospective cohort of 314 consecutive patients who underwent hepatectomy at Nanjing Drum Tower Hospital between January 2023 and December 2024 was included in the final analysis. The study protocol was approved by the Institutional Review Board of Nanjing Drum Tower Hospital (Approval No. 2023-382-03). Because of the retrospective nature of the study, the requirement for written informed consent was waived.

The inclusion criteria were as follows: (1) a clinical indication for hepatectomy and (2) availability of complete clinical documentation, including perioperative records and cognitive assessment data. Patients were excluded for any of the following reasons: in-hospital death within 72 h after surgery; preexisting psychiatric disorders, including dementia, depression, or delirium; a history of major neurological disease, such as ischemic stroke, hemorrhagic stroke, or severe head trauma; preoperative Montreal Cognitive Assessment (MoCA) score < 26; clinically overt hepatic encephalopathy (HE); prior neurosurgery; or significant visual or hearing impairment that interfered with cognitive evaluation.To assess whether the available sample size was adequate for the planned analysis, sample size estimation was performed using G*Power software, version 3.1.9.7. With an effect size of 0.5, a two-sided α of 0.05, and a statistical power of 0.80, the minimum required sample size was calculated. Allowing for potential attrition and incomplete assessment, a target sample size of 260 was considered acceptable.

### 2.2. Postoperative Cognitive Dysfunction (POCD) Assessment

POCD was assessed using the Montreal Cognitive Assessment (MoCA), a validated instrument covering multiple cognitive domains, including temporal and spatial orientation, executive function, calculation, naming, repetition, visuospatial ability, memory, and attention [19]. All assessments were performed by two trained evaluators at two time points: preoperatively (baseline) and on postoperative day 5. Inter-rater reliability was high (κ > 0.85).

Patients with a baseline MoCA score < 26 were excluded because this finding suggested preexisting cognitive impairment. In the analysis, POCD was defined as a decline of ≥3 points in the total MoCA score from baseline to postoperative day 5. Based on this criterion, patients were classified into the POCD and non-POCD groups for comparative analysis. Patients with clinically overt hepatic encephalopathy were not evaluated using the POCD scoring procedure. Importantly, patients with clinically overt hepatic encephalopathy (HE) were not evaluated using the POCD scoring procedure and were strictly excluded from the POCD classification analysis.

### 2.3. Covariates

The following variables were collected for analysis: demographic characteristics (sex, age, and educational level); liver function–related variables, including Child–Pugh classification and post-hepatectomy liver failure (PHLF); preoperative laboratory parameters, including C-reactive protein, albumin, and hemoglobin; nutritional status indicators, including sarcopenia; operative variables, including extent of hepatectomy and operative duration; lifestyle factors, including chronic alcohol consumption (defined as documented daily intake of ≥40 g of pure alcohol for men, or ≥20 g for women, sustained for at least 5 years); comorbidities, including diabetes mellitus; perioperative medication exposure, including opioid analgesics and sedative medications; and baseline psychosocial measures, including the Self-Rating Anxiety Scale (SAS) and Pittsburgh Sleep Quality Index (PSQI).

### 2.4. Data Collection

All clinical data were extracted from the electronic medical record system of Nanjing Drum Tower Hospital. Two investigators independently reviewed the medical charts, operative records, and perioperative documentation to ensure data accuracy and completeness. Any discrepancies were resolved through discussion with a senior investigator. The variables included in the analysis were selected based on the prior literature and clinical relevance, with particular attention to perioperative factors potentially associated with postoperative cognitive outcomes.

### 2.5. Statistical Analysis

Statistical analyses were performed using Zstats 1.0 and R software version 4.3.3. Continuous variables were assessed for normality before analysis. Intergroup comparisons were performed using Student’s *t* test, Mann–Whitney U test, or χ^2^ test, as appropriate. Variables with a *p* value < 0.05 in univariable analysis were entered into a multivariable logistic regression model using a stepwise selection strategy to identify variables independently associated with POCD. A nomogram was then constructed on the basis of the final multivariable model using the rms package, and internal validation was performed with 1000 bootstrap resamples. A two-sided *p*-value < 0.05 was considered statistically significant.

### 2.6. Ethical Considerations

The study was approved by the Institutional Review Board of Nanjing Drum Tower Hospital (Approval No. 2023-382-03). Owing to the retrospective nature of the study, the requirement for written informed consent was waived.

## 3. Results

### 3.1. General Characteristics

Among the 314 patients included in the final analysis, 86 (27.4%) were classified as having postoperative cognitive dysfunction (POCD) according to the revised diagnostic criterion. Comparative analysis showed significant differences between the POCD and non-POCD groups in several perioperative characteristics, including Child–Pugh classification, alcohol consumption, sarcopenia, preoperative hemoglobin level, operative duration, and pain score on postoperative day 1 (Table 1). Notably, consistent with the exclusion criteria, no patients with clinically overt hepatic encephalopathy were included in either the POCD or non-POCD groups, ensuring that the observed cognitive changes were distinct from overt HE.

### 3.2. Risk Factor Profiling

Univariable logistic regression identified several variables potentially associated with POCD, including Child–Pugh classification, alcohol consumption, sarcopenia, preoperative hemoglobin level, operative duration, and postoperative day 1 pain score. In the multivariable logistic regression analysis, Child–Pugh classification, alcohol consumption, sarcopenia, preoperative hemoglobin < 120 g/L, longer operative duration, and higher postoperative day 1 pain score were retained in the final model (Table 2). These findings suggest that POCD after hepatectomy may be associated with the combined effects of hepatic functional status, nutritional reserve, perioperative stress, and postoperative recovery burden.

### 3.3. Predictive Modeling POCD in Hepatectomy Patients

**Figure 1:**
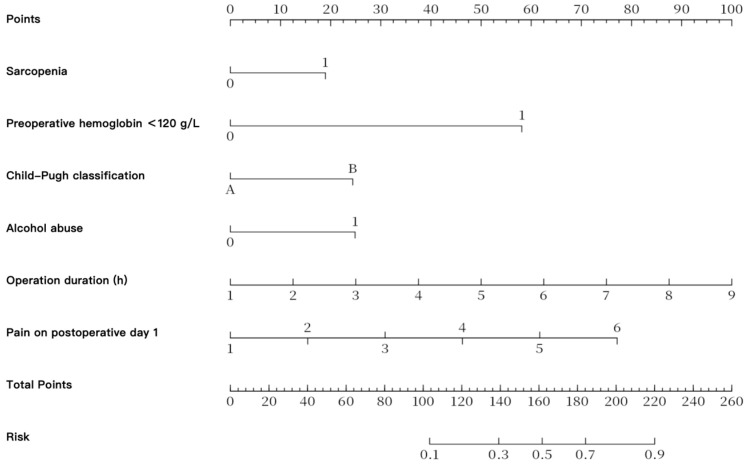
Nomogram for Predicting POCD Risk in Patients Undergoing Hepatectomy Based on Multivariate Regression.

A visual nomogram incorporating the six variables retained in the final multivariable model was developed (Figure 1), including Child–Pugh classification, alcohol consumption, sarcopenia, preoperative hemoglobin < 120 g/L, operative duration, and postoperative day 1 pain score. The nomogram assigns weighted scores to each variable according to its corresponding regression coefficient, allowing individualized estimation of POCD probability after hepatectomy.

**Figure 2:**
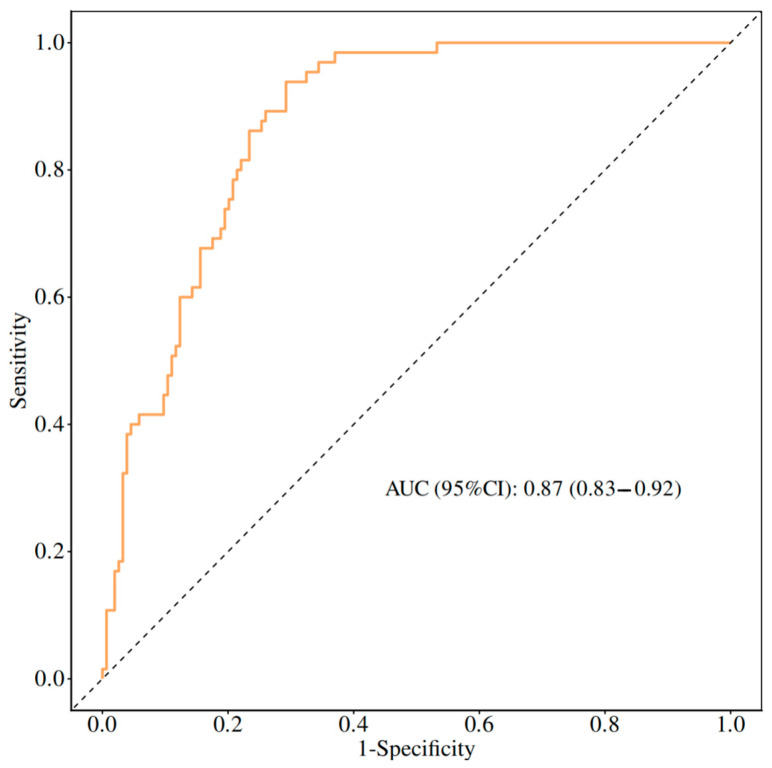
Receiver operating characteristic (ROC) curve of the nomogram for predicting postoperative cognitive dysfunction (POCD) in patients undergoing hepatectomy.

Model performance was evaluated using receiver operating characteristic (ROC) analysis. The area under the ROC curve (AUC) was 0.87 (95% confidence interval [CI]: 0.83–0.92), indicating good discriminative ability of the nomogram (Figure 2).

## 4. Discussion

This retrospective study showed that Child–Pugh classification, alcohol consumption, sarcopenia, preoperative hemoglobin < 120 g/L, operative duration, and postoperative day 1 pain score were retained in the final multivariable model and incorporated into the nomogram. Because of the retrospective design, these findings should be interpreted as associations rather than causal effects. Overall, the results suggest that postoperative cognitive outcomes after hepatectomy may reflect the combined influence of hepatic functional reserve, nutritional and hematologic status, perioperative stress exposure, and early postoperative recovery burden, rather than any single perioperative factor alone.

From a pathophysiological perspective, chronic alcohol consumption contributes not only to liver injury but also permits its metabolite, acetaldehyde, to disrupt the blood–brain barrier, activate neuroinflammatory pathways, and induce neuronal apoptosis [20]. Nutritional deficiencies commonly associated with chronic alcohol use may further aggravate neurological damage [21]. In the analysis, sarcopenia was retained in the final model, suggesting that impaired nutritional and functional reserve may be more closely associated with postoperative cognitive vulnerability than serum albumin alone in this cohort.

Similarly, hypoalbuminemia signifies both poor nutritional status and reduced antioxidant capacity, which may contribute to cognitive decline by impairing the transport of neurotrophic factors, increasing blood–brain barrier permeability, and facilitating the entry of pro-inflammatory cytokines into the central nervous system [22]. Although albumin was not retained in the revised multivariable model, this mechanism remains biologically relevant and may partly explain the relationship between nutritional impairment and postoperative neurocognitive decline.

A prolonged operative duration extends exposure to anesthetic agents, heightens the risk of hemodynamic instability, and exacerbates tissue ischemia–reperfusion injury. Collectively, these factors amplify the risk of neurological injury by promoting cumulative oxidative stress and inflammatory cascades [23,24]. In addition, postoperative day 1 pain score was also retained in the final model. This finding suggests that early postoperative stress burden may be relevant to short-term neurocognitive recovery, as poorly controlled pain may adversely affect sleep, mobilization, and overall physiologic stability. The retention of Child–Pugh classification in the final model further suggests that baseline hepatic functional reserve may also contribute to postoperative cognitive vulnerability in patients undergoing hepatectomy.

Preoperative hemoglobin < 120 g/L was another variable retained in the final model. Lower hemoglobin levels may reflect reduced oxygen-carrying capacity and limited physiologic reserve, thereby increasing susceptibility to perioperative stress. From a clinical perspective, these findings indicate that several variables associated with POCD may be potentially modifiable in routine perioperative management.

The present nomogram integrates six routinely available perioperative variables and showed good discriminative ability in internal validation. This suggests that a simple visual model based on commonly collected clinical data may help identify patients at higher risk of postoperative cognitive dysfunction after hepatectomy.

Future research should focus on validating the present nomogram in larger multicenter cohorts and exploring additional biomarkers that may further improve model performance. Integration of biochemical indicators, inflammatory markers, and more refined perioperative variables may help optimize risk stratification for postoperative cognitive dysfunction in patients undergoing hepatectomy. In addition, dynamic perioperative monitoring rather than single-time-point assessment may further enhance the accuracy and clinical applicability of future models.

This model may also have practical implications for perioperative care. Preoperative evaluation of nutritional status, correction of anemia when appropriate, management of alcohol-related exposure, and optimization of postoperative pain control may all be relevant components of a multidimensional strategy for patients considered at higher risk of POCD. Whenever feasible, minimally invasive techniques (e.g., laparoscopy) should be employed to reduce operative duration and surgical stress [25]. As operative duration can often be anticipated on the basis of procedure complexity, patients scheduled for more extensive or technically demanding resections may warrant closer perioperative neurocognitive surveillance. The nomogram may also support communication with patients and families by providing a more intuitive estimate of postoperative cognitive risk.

Collectively, these targeted measures address modifiable risk factors and align with the fundamental goals of enhanced recovery and personalized care in liver surgery patients [26]. Several issues still warrant further investigation. First, although the nomogram performed well in internal validation, external validation in independent cohorts remains necessary before broader clinical use. Second, as this was a retrospective observational study, residual confounding cannot be fully excluded. Third, future prospective studies with repeated cognitive assessments may provide a clearer understanding of the temporal pattern and clinical significance of postoperative cognitive decline after hepatectomy.

## 5. Limitations

The present study has several limitations that should be acknowledged. First, this was a single-center retrospective study, and therefore selection bias, residual confounding, and limited generalizability cannot be fully excluded. Second, although patients with clinically overt HE were excluded, we cannot entirely exclude the potential influence of subclinical hepatic dysfunction or minimal HE on neurocognitive outcomes, which may overlap with POCD symptoms. Third, we did not collect certain potentially relevant variables, such as blood ammonia, bilirubin levels, disease duration, or chronic respiratory conditions, which may also influence cognitive outcomes. Although chronic alcohol consumption was defined using sex-specific daily alcohol intake thresholds and a minimum duration of 5 years, it was analyzed only as a dichotomous variable. Therefore, more detailed information on cumulative alcohol dose, drinking patterns, and changes in alcohol exposure over time was unavailable. Fourth, no non-hepatectomy control group was included; therefore, it was not possible to determine whether the observed postoperative cognitive changes were specific to hepatectomy or reflected more general postoperative effects. Fifth, although patients with clinically overt hepatic encephalopathy were not included in the POCD scoring procedure, the potential influence of postoperative liver dysfunction on neurocognitive outcomes still warrants further investigation. Furthermore, while the nomogram shows good discrimination, its predictive accuracy and clinical utility require validation in multicenter, prospective cohorts. Finally, the use of conventional logistic regression, although clinically interpretable, does not account for potential non-linear relationships or interactions among predictors.

## 6. Conclusions

In this retrospective cohort of patients undergoing hepatectomy, Child–Pugh classification, alcohol consumption, sarcopenia, preoperative hemoglobin < 120 g/L, operative duration, and postoperative day 1 pain score were associated with POCD. The nomogram integrating these routinely available perioperative variables may provide a practical framework for individualized risk estimation and early identification of patients at higher risk of postoperative cognitive dysfunction.

## Figures and Tables

**Table 1:** Comparison of Baseline Characteristics Between POCD Group and Non-POCD Group (n = 314).

Variables	Total	no-POCD	POCD	χ^2^/t	P
	(n = 314)	(n = 228)	(n = 86)		
Gender				χ^2^ = 0.06	0.8
Male	212 (67.52)	153 (67.11)	59 (68.60)		
Female	102 (32.48)	75 (32.89)	27 (31.40)		
Age				χ^2^ = 4.98	0.083
<65	95 (30.25)	74 (32.46)	21 (24.42)		
65–75	180 (57.32)	131 (57.46)	49 (56.98)		
>75	39 (12.42)	23 (10.09)	16 (18.60)		
Educational Attainment				χ^2^ = 2.12	0.549
Illiterate	26 (8.28)	19 (8.33)	7 (8.14)		
Primary school	68 (21.66)	49 (21.49)	19 (22.09)		
Middle school	145 (46.18)	110 (48.25)	35 (40.70)		
Undergraduate and above	75 (23.89)	50 (21.93)	25 (29.07)		
Diabetes mellitus, n (%)				χ^2^ = 0.81	0.368
no	265 (84.39)	195 (85.53)	70 (81.40)		
yes	49 (15.61)	33 (14.47)	16 (18.60)		
Alcohol consumption, n (%)				χ^2^ = 8.67	0.003
no	152 (48.41)	122 (53.51)	30 (34.88)		
yes	162 (51.59)	106 (46.49)	56 (65.12)		
Preoperative Self-Rating Anxiety Scale (SAS) score	29.07 ± 7.31	29.07 ± 7.12	29.06 ± 7.84	t = 0.02	0.986
Preoperative Pittsburgh Sleep Quality Index (PSQI) score	7.54 ± 3.01	7.35 ± 2.87	8.05 ± 3.30	t = −1.85	0.066
Child–Pugh classification				χ^2^ = 6.05	0.014
A	232 (73.89)	177 (77.63)	55 (63.95)		
B	82 (26.11)	51 (22.37)	31 (36.05)		
Preoperative albumin				χ^2^ = 3.35	0.067
≥35 g/L	211 (67.20)	160 (70.18)	51 (59.30)		
<35 g/L	103 (32.80)	68 (29.82)	35 (40.70)		
Sarcopenia				χ^2^ = 4.16	0.041
No	275 (87.58)	205 (89.91)	70 (81.40)		
Yes	39 (12.42)	23 (10.09)	16 (18.60)		
Preoperative hemoglobin				χ^2^ = 7.14	0.008
<120 g/L	67 (21.34)	40 (17.54)	27 (31.40)		
≥120 g/L	247 (78.66)	188 (82.46)	59 (68.60)		
Operation duration (h)	2.83 ± 1.10	2.72 ± 1.01	3.12 ± 1.27	t = −2.63	0.01
Pain score on postoperative day 1	3.34 ± 1.23	3.14 ± 1.23	3.86 ± 1.08	t = −4.75	<0.001
Use of opioid analgesics and sedative medications, n (%)				χ^2^ = 0.54	0.461
No	32 (10.19)	25 (10.96)	7 (8.14)		
Yes	282 (89.81)	203 (89.04)	79 (91.86)		
Post-hepatectomy liver failure (PHLF), n (%)				χ^2^ = 0.06	0.814
No	279 (88.85)	202 (88.60)	77 (89.53)		
Yes	35 (11.15)	26 (11.40)	9 (10.47)		
Extent of hepatectomy, n (%)				χ^2^ = 5.08	0.079
Major hepatectomy (H-R, H-L)	32 (10.19)	27 (11.84)	5 (5.81)		
Intermediate hepatectomy (sectionectomy)	235 (74.84)	172 (75.44)	63 (73.26)		
Minor hepatectomy (segmentectomy, non-anatomical resection)	47 (14.97)	29 (12.72)	18 (20.93)		
Preoperative C-reactive protein (CRP) (mg/L)	4.20 (2.90, 7.10)	4.15 (3.00, 7.10)	4.25 (2.80, 7.53)	Z = −0.15	0.884

Note: Alcohol consumption was defined as chronic alcohol abuse, specifically referring to documented daily intake of ≥40 g of pure alcohol for males or ≥20 g for females, sustained for at least 5 years, rather than occasional drinking.

**Table 2:** Multivariable Logistic Regression Analysis of Variables Associated with Postoperative Cognitive Dysfunction in Patients Undergoing Hepatectomy.

Variable	β	S.E	Z	*p* Value	OR (95%CI)
Intercept	−0.53	0.10	−5.18	<0.001	—
Child–Pugh classification					
A					Reference
B	0.58	0.27	2.13	0.033	1.78 (1.05–3.02)
Sarcopenia					
No					Reference
Yes	0.71	0.35	2.01	0.044	2.04 (1.02–4.08)
Preoperative hemoglobin < 120 g/L					
No					Reference
Yes	1.58	0.45	3.53	0.003	2.24 (1.27–4.24)
Alcohol consumption					
No					Reference
Yes	0.76	0.26	2.92	0.004	2.15 (1.28–3.59)
Operative duration (h)	0.32	0.11	2.82	0.005	1.38 (1.10–1.72)
Postoperative day 1 pain score	0.49	0.11	4.47	<0.001	1.63 (1.32–2.03)

Abbreviations: OR: Odds Ratio; CI: Confidence Interval; S.E, standard error.

## Data Availability

The datasets generated and/or analyzed during the current study are available from the corresponding author on reasonable request.
